# Antioxidant, Antiglaucoma, Anticholinergic, and Antidiabetic Effects of Kiwifruit (*Actinidia deliciosa*) Oil: Metabolite Profile Analysis Using LC-HR/MS, GC/MS and GC-FID

**DOI:** 10.3390/life13091939

**Published:** 2023-09-20

**Authors:** Eda Mehtap Ozden, Zeynebe Bingol, Muzaffer Mutlu, Hasan Karagecili, Ekrem Köksal, Ahmet C. Goren, Saleh H. Alwasel, İlhami Gulcin

**Affiliations:** 1 Department of Chemistry, Faculty of Science, Ataturk University, Erzurum 25240, Türkiye; edamehtap3@gmail.com; 2 Department of Medical Services and Techniques, Tokat Vocational School of Health Services, Gaziosmanpasa University, Tokat 60250, Türkiye; zeynep.bingol196@gmail.com; 3 Vocational School of Applied Sciences, Gelisim University, Istanbul 34315, Türkiye; muzaffermutlu@hotmail.com; 4 Department of Nursing, Faculty of Health Sciences, Siirt University, Siirt 56100, Türkiye; 5 Department of Chemistry, Faculty of Science and Arts, Erzincan Binali Yildirim University, Erzincan 24100, Türkiye; koksalekrem@gmail.com; 6 Department Chemistry, Faculty of Sciences, Gebze Technical University, Kocaeli 41400, Türkiye; acgoren@gtu.edu.tr; 7 Department of Zoology, College of Science, King Saud University, Riyadh 11362, Saudi Arabia; salwasel@ksu.edu.sa

**Keywords:** *Actinidia deliciosa*, polyphenol, kiwifruit oil, antioxidant activity, LC-HRMS, GC-MS, enzyme inhibition

## Abstract

Determining the antioxidant abilities and enzyme inhibition profiles of medicinally important plants and their oils is of great importance for a healthy life and the treatment of some common global diseases. Kiwifruit (*Actinidia deliciosa*) oil was examined and researched using several bioanalytical methods comprehensively for the first time in this research to determine its antioxidant, antiglaucoma, antidiabetic and anti-Alzheimer’s capabilities. Additionally, the kiwifruit oil inhibitory effects on acetylcholinesterase (AChE), carbonic anhydrase II (CA II), and α-amylase, which are linked to a number of metabolic illnesses, were established. Furthermore, LC-HRMS analysis was used to assess the phenolic content of kiwifruit oil. It came to light that kiwifruit oil contained 26 different phenolic compounds. According to the LC-HRMS findings, kiwifruit oil is abundant in apigenin (74.24 mg/L oil), epigallocatechin (12.89 mg/L oil), caryophyllene oxide (12.89 mg/L oil), and luteolin (5.49 mg/L oil). In addition, GC-MS and GC-FID studies were used to ascertain the quantity and chemical composition of the essential oils contained in kiwifruit oil. Squalene (53.04%), linoleoyl chloride (20.28%), linoleic acid (2.67%), and palmitic acid (1.54%) were the most abundant compounds in kiwifruit oil. For radical scavenging activities of kiwifruit oil, 1,1-diphenyl-2-picryl-hydrazil (DPPH^•^) and 2,2′-azino-bis(3-ethylbenzthiazoline-6-sulfonic acid) (ABTS^•+^) radicals scavenging techniques were examined. These methods effectively demonstrated the potent radical scavenging properties of kiwifruit oil (IC_50_: 48.55 μg/mL for DPPH^•^, and IC_50_: 77.00 μg/mL for ABTS^•+^ scavenging). Also, for reducing capabilities, iron (Fe^3+^), copper (Cu^2+^), and Fe^3+^-2,4,6-tri(2-pyridyl)-S-triazine (TPTZ) reducing abilities were studied. Moreover, kiwifruit oil showed a considerable inhibition effect towards hCA II (IC_50_: 505.83 μg/mL), AChE (IC_50_: 12.80 μg/mL), and α-amylase (IC_50_: 421.02 μg/mL). The results revealed that the use of kiwifruit oil in a pharmaceutical procedure has very important effects due to its antioxidant, anti-Alzheimer, antidiabetic, and antiglaucoma effects.

## 1. Introduction

Natural supplies obtained from plants are being utilized more and more in a variety of industries, such as food, cosmetics, and pharmaceuticals. Due to the role that plant-based goods play in preserving human health, enthusiasm for the biological activities of these items has grown over the past few years [1]. Fruits with high biological activity have been advocated for in several studies as a means of avoiding illness and therapy. A healthy diet should include eating vegetables and fruits regularly [2]. Polyphenols are secondary plant metabolic byproducts found in most vegetables and fruits. Plants often produce polyphenols to protect themselves against internal and external stresses. Also, polyphenols, which have a significant effect on the organoleptic qualities of plants, are frequently used, especially in food and cosmetic formulations [3,4]. In addition to being good for the ecosystem, plants function as living chemical factories that generate a variety of secondary metabolites. In terms of cosmetic, food, and pharmaceutical sectors, medicinal plants are of the utmost importance [5].

Oxidative stress is brought on by unbalanced ROS and antioxidant systems. Oxidative stress disrupts many cellular processes and causes a number of pathological conditions where the body’s antioxidative defenses are overwhelmed by ROS, resulting in tissue damage, stimulated cell death, and the oxidative alteration of the biological macromolecules mentioned above [6]. ROS are mostly caused by normal aerobic metabolism and environmental conditions such as air and water pollution, smoke, radiation, stress, malnutrition and excessive and unconscious drug use [7]. To counteract the negative consequences of ROS and free radicals, antioxidants must be present in biological systems, though in small amounts [8]. The oxidative deterioration caused by ROS and free radicals in living organisms is therefore prevented by antioxidants. By supplying phenolic antioxidants from natural sources, they can reduce the negative impacts of ROS and free radicals. They are ascribed to these plants’ secondary metabolites, notably to the phenolic chemicals that make up a significant portion of these metabolites, through methods like the elimination of free radicals and the inhibition of particular metabolic enzymes and processes in cells. In particular, plants’ antioxidant capabilities, besides the biological functions of phenolic chemicals originating from plants, have been thoroughly investigated. Moreover, plants exhibit great therapeutic value for various chronic illnesses because of their physiologically functioning regulator content [9].

The genus Actinidia, which includes the kiwi fruit, is part of the Actinidiaceae family. There are several additional names for kiwifruit, but only two of the 76 species in the genus Actinidia-*Actinidia chinensis* and *Actinidia deliciosa* are grown for commercial purposes [10]. Bioactive phytochemicals found in kiwifruit of all species and varieties also include non-starch polysaccharides, minerals, carotenoids, chlorophylls and organic acids. Antioxidative, antihypertensive, anticancer, neuroprotective, antiobesity and the promotion of gut health are some of the health-promoting properties of kiwifruit [11]. After being extracted using water vapor distillation, the chemical ingredients of *A. deliciosa* caudexes volatile oil was isolated and determined using GC-MS. Nineteen different chemical molecules in total, making up 97.37% of the total, were found [12].

Antioxidants are a class of chemicals, synthetic or natural, which are clearly associated with great health benefits and lower risks of various age-related diseases. In this context, it was reported that the undesirable consequences of synthetic antioxidants, such as BHT and BHA, have also been studied and are widely recognized. However, because of their harmful and carcinogenic effects on living, artificial antioxidant usage has been curbed. Despite that, significant sources of natural antioxidants are medicinal plants, which have been the focus of several pieces of research to date. The number and quantity of phenols in medicinal plants are substantial. The major natural sources of antioxidants in the average person’s diet are fruits, vegetables and grains. Phenolic compounds protect against a large spectrum of degenerative conditions like cataracts, heart disorders, carcinoma, hypercholesterolemia, diabetes, and rheumatoid arthritis [13]. As a result of dietary plant components, the vascular endothelium performs better, reducing the risk of diabetes, glaucoma, Alzheimer’s disease (AD) and other cardiovascular illnesses as well as cancer [14].

Carbonic anhydrases (CAs) are metalloenzymes that contribute to the pH maintenance in the cells, catalyzing the hydration of water and carbon dioxide (CO_2_) to protons (H^+^) and bicarbonate ions (HCO_3_^−^) reversibly [15]. High intraocular pressure (IOP) is the primary component in the multifactorial visual disease known as glaucoma, which may result in blindness. After topical application, CA inhibitors (CAIs) like acetazolamide and dorzolamide are efficient in lowering IOP; however, these treatments have a variety of adverse effects, necessitating the development of novel therapeutic strategies [16]. In fact, CA isoforms provide a large medicinal target, and the creation of isoenzyme-selective CAIs is an excellent approach for producing a medication with the fewest adverse effects [17]. Several potent CAIs, including acetazolamide, methazolamide, ethoxzolamide, dichlorphenamide, dorzolamide, brinzolamide, etc., have been used clinically for decades as diuretics, antiglaucoma medicines and antiepileptics [18,19]. It is preferable to use inhibitors with topical action that originates from nature to prevent these unwanted side effects. As a result, we draw the attention of glaucoma investigators to the use of kiwifruit oil in therapy.

Insulin is produced or used improperly in diabetes mellitus (DM) and endocrine system metabolic disorders. The 90% to 95% of instances of diabetes are type-2 DM (T2DM) or non-insulin-dependent diabetic mellitus. An unusually high fasting and postprandial blood glucose level is a defining feature of T2DM. This disease is ascribed to insulin insufficiency, which results from diminished secretion from impaired beta cells, as well as insulin resistance, in which muscle and fat cells lose their sensitivity to insulin. Less glucose enters the cells as a consequence of decreased glucose intake [20]. Plants and plant-derived substances are used to treat AD, but they also have an impact on the biochemical processes involved in problems from diabetes. In the past, drugs with plant origins, like metformin, were used to treat both DM and low blood sugar [21]. Likewise, oligosaccharide and polysaccharide molecules are hydrolyzed by the enzymes α-amylase and α-glycosidase, which are secreted from small intestinal cells into monosaccharide units like glucose and fructose. Inhibitors of digestive enzymes have been shown to reduce the absorption of dietary carbohydrates and to control T2DM and postprandial hyperglycemia. They successfully lower postprandial polysaccharide units, particularly glucose, in T2DM as a result. Therefore, T2DM and hyperglycemia can be treated using digestive enzyme inhibitors (DEIs) that can be found in natural sources. Because they decrease intestinal glucose absorption, DEIs have an antidiabetic effect and lower blood sugar levels. As a result, DEIs and oligosaccharides compete for binding to the enzyme’s active site. One common inhibitor for this type of inhibition is acarbose [22].

Alzheimer’s disease (AD) is a neurodegenerative ailment that advances quickly, resulting in behavioral modifications, forgetfulness, mental decline and linguistic deficits. Acetylcholinesterase (AChE) degrades acetylcholine (ACh) to produce acetate (CH_3_COO^−^) and choline (Ch). The inhibition of AChE has recently been proven to play a crucial role in enhancing cholinergic transmission in the brain as well as reducing amyloid-β aggregation and the production of neurotoxic fibrils in AD [23]. In accordance with the cholinergic theory, neurotransmitter ACh levels in the brain are lowered in AD patients, leading to memory impairment [24]. ACh is hydrolyzed by AChE into CH_3_COO^−^ and Ch, while butyrylcholinesterase (BChE) changes butyrylcholine (BCh) into butyrate and Ch. Because of the loss of neurons in the central nervous system, AD is classified as a neurodegenerative condition with gradual progression and functional impairment. Acute consumption of the neurotransmitter ACh is recognized to have a major role in the pathogenesis of AD. Remarkably common cholinesterase inhibitors include tacrine, galantamine, donepezil and rivastigmine. Many of the negative effects of these therapeutic inhibitors result in treatment cessation. Constipation, hepatotoxicity, nausea, vomiting and diarrhea are among the most serious negative consequences [25]. In scientific studies in medical science, particularly for the amelioration of AD, natural compounds like AChE inhibitors (AChEIs) were frequently used. Phenolic compounds were also discovered to be AChEIs and to be the first drugs in treating AD [26]. 

It is known that phenolic compounds, secondary plant metabolites, which have antioxidant effects, are very effective in the development of treatments for the above-mentioned health problems. Plant-derived oils, extracts or components, including kiwi oil, can be used to treat the above-mentioned health problems [8,13].

In the present research, we assessed the chemical constitution, antioxidant, anti-Alzheimer, antiglaucoma and antidiabetic properties of kiwifruit oil. Employing LC-HRMS and GS-MS/FID, we also estimated the polyphenol and essential oil composition in kiwifruit oil.

## 2. Materials and Methods

### 2.1. Chemicals

α-Tocopherol, Trolox, butylated hydroxyanisole (BHA), 1,1-diphenyl-2-picrylhydrazyl (DPPH), and butylated hydroxytoluene (BHT) were all bought from Sigma-Aldrich Chemie GmbH (Steinheim, Germany). We bought the following chemicals from Sigma-Aldrich: 2,2′-azino-bis(3-ethylbenzthiazoline-6 sulfonic acid) (ABTS) ascorbic acid, fumaric acid, chlorogenic acid, caffeic acid, naringin, vanillic acid, syringic acid, rutin, rosmarinic acid, p-coumaric acid, salicylic acid, quercetin, luteolin, naringenin, and chrysin. We also obtained hyperoside, luteolin 7-glycoside, orientin, (+)-trans-taxifolin, quercitrin, hispidulin, apigenin, hederagenin, and acacetin from TRC in Canada. The companies HWI Analytik GMBH (Rheinzaberner, Germany) and Carbosynth Ltd. (Berkshire, UK) provided the verbascoside and luteolin-7-rutinoside, respectively. The supplier of hesperidin was J & K Co., Ltd. (Seoul, Republic of Korea). We bought penduletin, dihydrokaempferol and iso-sakuranetin from Phytolab (Vestenbergsgreuth, Germany). From EDQM CS, apigenin 7-glucoside was obtained. We bought myricetin from Carl Roth GmbH & Co. (Karlsruhe, Germany) European Pharmacopoeia provided the nepetin, while Supelco (Bellefonte, PA, USA) provided the caffeic acid phenethylester.

### 2.2. Preparation of Kiwifruit (A. deliciosa) Oil

Kiwifruits (*A. deliciosa*) were bought from a nearby market. Steam distillation was used to create kiwifruit oil. This technique consists of numerous phases of continuous distillation that separate the oils utilizing steam as the stripping gas. The plant receives direct steam application. The combination of vapors is gathered and concentrated to produce liquid with two separate layers of water and oil. The hydrophobic molecules found in essential oils make up the top portion of these layers, while the hydrophilic compounds found in a hydrolysate or hydrosol make up the lower portion. Cohobating allows for the recovery of any polar chemicals that are still present in the water. The steam intake of the steam distillation apparatus was positioned over a grid of dried kiwifruit. For almost two hours, steam was provided to the appliance. Using a chiller to condense the combination, which included water vapor and volatile essential oils, was then gathered in the collecting container. Because water and kiwifruit oil have different densities, the kiwifruit oil collected in the collecting container separated from the water layer and was then removed.

### 2.3. Polyphenolic Composition Using LC-HRMS Analysis

LC-HRMS tests were conducted using a Troyasil C18 column (150 × 3 mm i.d., 3 µm particle size) (Istanbul, Türkiye) and a Thermo Orbitrap Q-Exactive mass spectrometer (Thermo Fisher Scientific Inc., Waltham, MA, USA). The mobile phases A and B were made up, respectively, of 1% formic acid–water and 1% formic acid–methanol. The gradient programs were 50% A and 50% B for 0–1 min, 100% B for 1–6 min, and 50% A and 50% B for 6–10 min. The column temperature was set at 22 °C, and the mobile phase flow rate was 0.35 mL/min. The temperature and relative humidity were set at 22.0 ± 5.0 °C and 50 ± 15%, respectively [27]. Based on previous findings and information from the literature, it was determined that an acidified methanol and water gradient was the best solvent system for producing sufficient ionization abundance and separating compounds in HPLC. We selected the ESI source for the applicable approach since it offers one of the best ionizations for tiny and relatively polar molecules. The instrument high-decision technique was used to survey the ions with m/z values between 85 and 1500 [27]. Retention times and HRMS data were compared to those of standard compounds (95–99% pure; see Section 2.1) in order to identify the different compounds. For LC-HRMS measurements, dihydrocapsaicin (purity 95%) was utilized as an internal standard in order to lessen the repeatability issue brought on by external factors, such as ionization repeatability. Table 1 includes a list of the mass parameters for each target chemical. The LC-HRMS technique, uncertainty evaluation approach and confirmation criteria for phenolics were all previously covered in great depth [27,28].

Liquid–liquid extraction was used to obtain the kiwifruit oil in order to analyze the species’ secondary metabolite profile. Mobile phase B (1% formic acid in methanol) was used to dissolve 100 mg of kiwifruit oil in a 4 mL volumetric flask, which was then placed in an ultrasonic bath for 10 min. After that, mobile phase B was used to regulate the volume of 100 μL of the internal standard, which was a methanol–dihydrocapsaicin solution. A 0.45 μm Millipore Millex-HV filter was used to filter the final mixture before it was put (1 mL) into an autosampler vial that had been sealed. From there, 2 μL of the specimen was then examined using liquid chromatography for each run. Throughout the experiment, 15 °C was maintained for the samples in the autosampler. The composition of various aromatic compounds and essential oils was accurately analyzed qualitatively using GC-MS.

Analytical standards of the target chemicals employing negative or positive ions were used to validate the LC-HRMS technique. These standards are presented in Table 1. The internal standard utilized was dihydrocapsaicin. Limits of detection (LOD), limit of quantification (LOQ), reproducibility, recovery, and linearity were the characteristics used to validate the method. Using the following equation, the LODs of the approach for distinct substances were determined: LOD or LOQ = κSD*a*/*b*, where LOQ is 3 and κ = 3 for LOD. Aside from that, *b* stands for the slope and SD*a* stands for the standard deviation of the intercept. In earlier works, it was described in detail how the applied method’s validation process worked and how to quantify uncertainty [28].

### 2.4. GC/MS and GC-FID Analyses of Kiwifruit Oil and Essential Oil Isolation

The essential oil was kept at 4 °C until the GC-MS/FID computations, and the extract was dried over anhydrous CaCl_2_. The oil yield was 1.52%. On a DB-5 capillary column (60 m × 0.25 mm, 0.25 mm film thickness) with helium as the carrier gas (0.8 mL/min), GC-MS analysis was performed using a Thermo Scientific Trace GC 1310 linked to a Thermo TSQ 9610 MS system. After being held at 80 °C for 10 min, the GC oven’s temperature was scheduled to rise to 280 °C at a rate of 4 °C per minute, and it remained at 280 °C for 5 min. It was changed to a 1:20 split ratio. The injector was adjusted at a temperature of 250 °C. At a mass of 70 eV, the spectra were captured. The *m*/*z* 35–650 was the mass range. A Thermo Scientific Trace GC 1310 equipment was used to conduct the GC-FID analysis. The FID detector was held at a temperature of 280 °C. Parallel auto-injection was carried out twice on the same column with the same operating settings in order to achieve the same elution sequence as GC-MS. From the FID chromatograms, the relative proportion of the isolated chemicals was determined [29]. When calculating the Kovats Indices (KIs), alkanes were employed as reference points. Authentic samples, the NIST and Wiley spectra, as well as information from the literature, were used to compare retention durations and mass spectra, which helped identify the compounds [30,31,32].

### 2.5. Reducing Ability Assays

Using the Fe^3+^(CN^−^)_6_ complex reduction technique, the Fe^3+^ reduction potential of kiwifruit oil was ascertained. The addition of ferric ions (Fe^3+^) to the reduced product leads to the formation of the intense Perl’s Prussian blue complex, Fe_4_[Fe(CN^−^)_6_]_3_, which has a maximum absorbance at 700 nm [33]. For this, 2.5 mL of phosphate buffer (pH 6.6, 0.2 M), 2.5 mL of [K_3_Fe(CN)_6_] solutions (1%) and different quantities of kiwifruit oil were conveyed to test tubes. Following a vortex, the test solution was incubated for 25 min at 50 °C. After that, 2.5 mL of 10% trichloroacetic acid was included. After that, 0.5 mL of FeCl_3_ (0.1%) and 2.5 mL of distilled water were combined with 2.5 mL of the top layers of the mixtures. A 700 nm spectrophotometric measurement was performed to determine the absorbance of the kiwifruit oil’s reducing effects. The essential experimental methods using the methodology of Apak et al. were carried out [34]. According to a previous investigation, the absorbance of kiwifruit oil’s capacity to reduce Cu^2+^ was identified. To do this, test tubes containing kiwifruit oil at different amounts (10–30 μg/mL) were transferred along with 0.25 mL of CuCl_2_ solution (10 mM), 0.25 mL of ethanolic neocuproine solution (7.5 × 10^−3^ M), and 250 μL NH_4_Ac buffer solution (1.0 M). After 30 min of incubation, the total volume was raised to 2 mL with distilled water, and their absorbance levels were measured at 450 nm. The Fe^3+^-TPTZ complex’s capacity to reduce the ability of kiwifruit oil was also carried out in accordance with a prior study [35]. For this, 2.25 mL of newly made TPTZ solution (10 mM in 40 mM HCl) was added together with 2.25 mL of 20 mM FeCl_3_ solution to 2.5 mL of freshly made acetate buffer (0.3 M, pH 3.6). Then, various kiwifruit oil concentrations were added and incubated for 25 min at 37 °C. Spectrophotometric measurements at 593 nm were then made to determine the kiwifruit oil’s absorbance and reducing power. The outcomes of all studies using reducing capabilities were calculated as the arithmetic mean of their three repeats.

### 2.6. Radical Scavenging Assays

Using the Blois technique and the DPPH radical, the ability of kiwifruit oil to scavenge radicals was assessed [36]. In a nutshell, 1 mL of 0.1 mM DPPH^•^ solution produced in ethanol was poured into the kiwifruit oil at various quantities (10–30 μg/mL). The values for the absorbance were set at 1.00 ± 0.200. After that, a solution of 0.5 mL of the kiwifruit extract is added to 3 mL of the DPPH working mixture, which was then blended and left for 30 min in the dark. In the presence of an antioxidant in the reaction media, the purple hue vanishes. The maximum absorption wavelength of a freshly made DPPH radical solution is 517 nm. Sample absorbance was evaluated at 517 nm, and the studies were performed in 3 duplicates. The reaction mixture known as the blank is devoid of test substances [37].

The radical cation ABTS^•+^ was first produced by oxidizing an aqueous mixture of ABTS (7.0 mM) with oxidants of K_2_S_2_O_8_ (2.5 mM). The absorbance value of the control solution was set to 0.750 ± 0.025 at 734 nm by diluting ABTS^•+^ mixture with a phosphate buffer (0.1 M, pH 7.4). After that, various quantities (10–30 μg/mL) of ABTS^•+^ solution were poured into 3 mL of kiwifruit oil. At 734 nm, the ABTS^•+^ absorbance that remained apparent after 30 min was recorded [38].

The following applied formula, RSC: (%) = (1 − A_c_/A_s_) × 100, where A_c_ and A_s_ are the absorbance rates of the control and sample, was used to determine the radical scavenging potential (RSC) of kiwifruit oil. Moreover, the IC_50_ was calculated from the graphs and was given as µg/mL [37].

### 2.7. Acetylcholinesterase Inhibition Assay

Using Ellman’s approach [39], kiwifruit oil’s cholinergic enzyme inhibitory abilities were assessed, as well as kiwifruit oil’s inhibitory effects on AChE from *Electrophorus electricus*, according to prior research. As substrates, 5,5′-dithio-bis-(2-nitrobenzoic acid) (DTNB) and acetylthiocholine iodide (AChI) were both utilized. The following ingredients were combined in a test tube: 1 mL of Tris/HCl buffer (1.0 M, pH 8.0), 10 μL of kiwifruit oil at various concentrations, and 50 μL AChE. 50 μL of DTNB mixture (0.5 mM) was then conveyed after the specimen had been incubated at 25 °C for 15 min. Afterward, 50 μL of AChI solution (10 mM) was added to initiate the process. The absorbance at 412 nm was then measured [40].

### 2.8. α-Amylase Inhibition Assay

The Xiao technique [41] was used to assess the kiwifruit oil’s capability to inhibit α-amylase utilizing a starch substrate. Firstly, 1 g of starch was distributed in 50 mL of a 0.4 M NaOH solution, which was then heated for 20 min at 80 °C. After cooling, distilled water was used to regulate the pH to 6.9 and the volume to 100 mL. Then, 5 µL of the kiwifruit oil solution and 35 µL of the starch solution, 35 µL of phosphate buffer with a pH of 6.9, were combined. A 20 µL solution of α-amylase was added, and the mixture was incubated at 37 °C for an additional 20 min. Absorbance was evaluated at 580 nm after the process was finished through the adjoined 50 µL of HCl (0.1 M).

### 2.9. hCA II Inhibition Assay

As previously revealed, CA II isoenzymes were separated and purified utilizing human blood samples and Sepharose-4B-L-Tyrosine sulfanilamide affinity chromatography [42]. Following the purification of the enzymes, the protein concentrations were assessed at 595 nm using Bradford’s technique [43]. Utilizing a spectrophotometer (Shimadzu, Kyoto, Japan, UVmini-1240 UV-VIS) and the procedure of Verpoorte et al. [44]. an esterase activity experiment was performed at 348 nm. Acetazolamide (AZA) was utilized as a reference standard.

### 2.10. Determination of IC_50_ Value

The enzyme inhibition effects of kiwifruit oil were assessed using IC_50_ values. The graphs showing the enzyme activity as a function of increasing kiwifruit oil concentrations were used to determine IC_50_ values [45].

### 2.11. Statistical Analysis

Each experiment is repeated three times. The results are given as mean ± SD. The one-way ANOVA was followed up with Tukey’s post hoc test; significant differences were considered to have a value of *p* < 0.05.

## 3. Results

### 3.1. Polyphenolic Composition of Kiwifruit Oil

Stability, accuracy, linearity, selectivity, recovery and matrix influence of the analytes were evaluated to validate the LC-HRMS assay [46]. Kiwifruit oil was used in this investigation to identify 26 phenolic components. The LC-HRMS findings revealed that kiwifruit oil has abundant apigenin (74.24 mg/L oil), epigallocatechin (12.89 mg/L oil), caryophyllene oxide (12.89 mg/L oil), luteolin (5.49 mg/L oil), salicylic acid (4.84 mg/L oil), ascorbic acid (4.57 mg/L oil), quillaic acid (4.57 mg/L oil), rutin (4.54 mg/L oil) and naringenin (3.62 mg/L oil).

**Figure 1:**
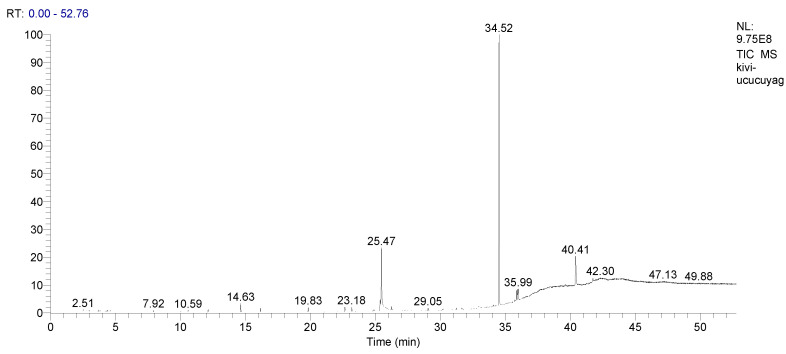
GC-MS analysis of kiwifruit (*Actinidia deliciosa*) oil and their identified % ratios.

The relative details of the aromatic components of kiwifruit oil are shown in Table 2. Six volatile components in kiwifruit oil samples were found in the current investigation. Among them, squalene (53.04%), linoleoyl chloride (20.28%), linoleic acid (2.67%), and palmitic acid (1.54%) were the most abundant compounds in kiwifruit oil (Table 2 and Figure 1).

### 3.2. Reducing Ability of Kiwifruit Oil

**Figure 2:**
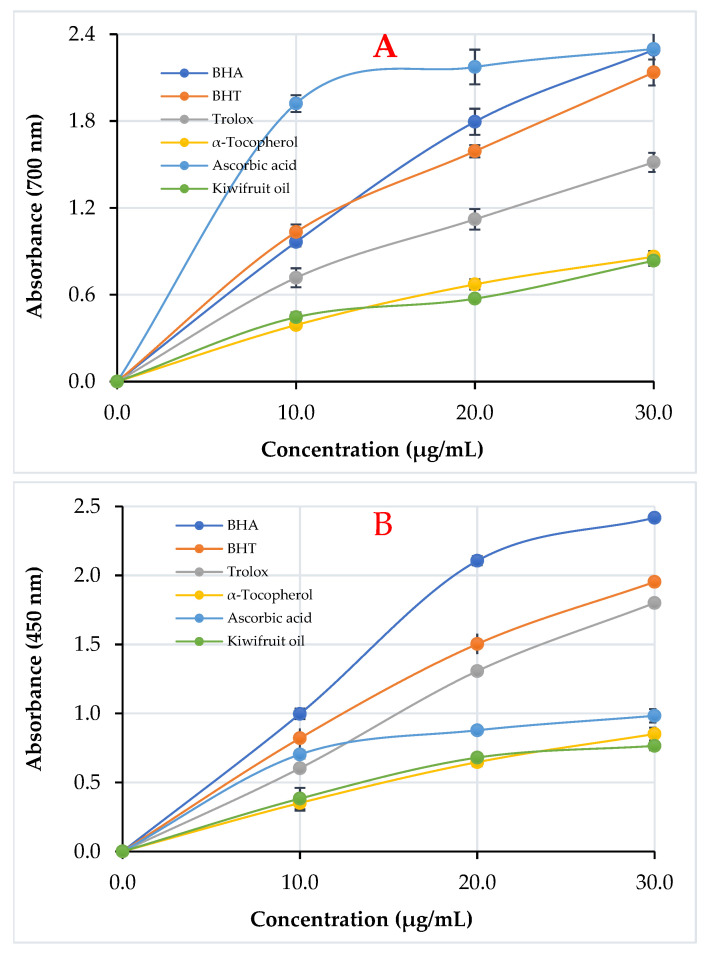

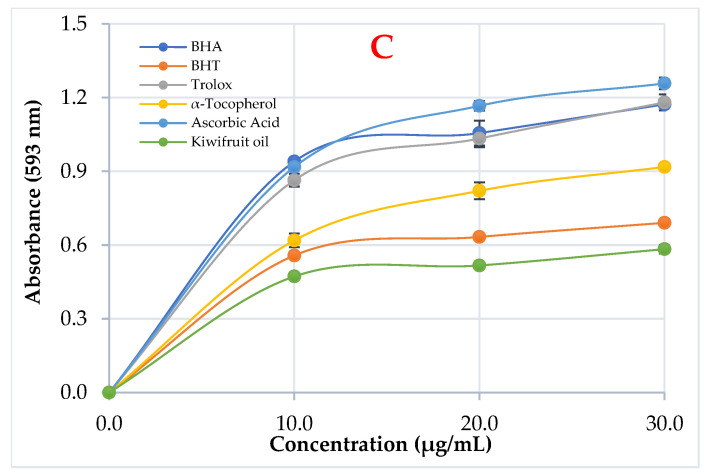
Ferric ions (Fe^3+^)-reducing (**A**), cupric ions (Cu^2+^)-reducing (**B**), and Fe^3+^-TPTZ complex-reducing (**C**) ability of kiwifruit (*A. deliciosa*) oil and standards.

In the Fe[Fe(CN^–^)_6_]_3_, Fe^3+^-TPTZ, and Cu^2+^ reduction experiment, kiwifruit (*A. deliciosa*) oil revealed a powerful and efficient reducing ability. To determine the reducing power of kiwifruit oil, a Fe^3+^–Fe^2+^ conversion experiment was first carried out (Figure 2A and Table 3). At 30 µg/mL, kiwifruit oil and references showed the potential Fe^3+^ reducing as follows: ascorbic acid (2.298 ± 0.086, r^2^: 0.9659) ≥ BHA (2.292 ± 0.012, r^2^: 0.9993) ≥ BHT (2.136 ± 0.090, r^2^: 0.9957) > Trolox (1.514 ± 0.066, r^2^: 0.9963) > α-tocopherol (0.862 ± 0.038, r^2^: 0.9996) ≥ kiwifruits oil (0.835 ± 0.035, r^2^ = 0.9723) ≥. The increased absorbance is due to complex formation and an improved reduction capacity (Figure 2A).

Figure 2B, C and Table 3 provide an overview of the investigation into kiwifruit oil’s Fe^3+^-TPTZ and Cu^2+^-reducing properties in addition to its Fe^3+^-reducing action. At the analyzed concentrations, kiwifruit oil had nearly high absorption values. The following is the order of the standards, kiwifruit oil, and reduced Cu^2+^ ions at a concentration of 30 μg/mL: (Figure 2B): BHA (2.418 ± 0.018, r^2^: 0.9887) > BHT (1.953 ± 0.045, r^2^: 0.9998) > Trolox (1.800 ± 0.096, r^2^: 0.9974) > ascorbic acid (0.983 ± 0.048, r^2^: 0.9822) > α-tocopherol (0.851 ± 0.046, r^2^: 0.9994) > kiwifruit oil (0.765 ± 0.031, r^2^: 0.9978).

In this reduction experiment, the kiwifruit oil demonstrated an efficient reducing potential (Table 3 and Figure 2C). The FRAP-reducing ability was decreased in the following order in test items comprising kiwifruit oil and standards: ascorbic acid (1.257 ± 0.024, r^2^: 0.9869) > Trolox (1.180 ± 0.032, r^2^: 0.9732) ≥ BHA (1.172 ± 0.014, r^2^: 0.9605) > α-tocopherol (0.918 ± 0.011, r^2^: 0.9904) > BHT (0.690 ± 0.008, r^2^: 0.9645) > kiwifruit oil (0.583 ± 0.017, r^2^: 0.9525).

### 3.3. Radicals Scavenging Effect of Kiwifruit Oil

**Figure 3:**
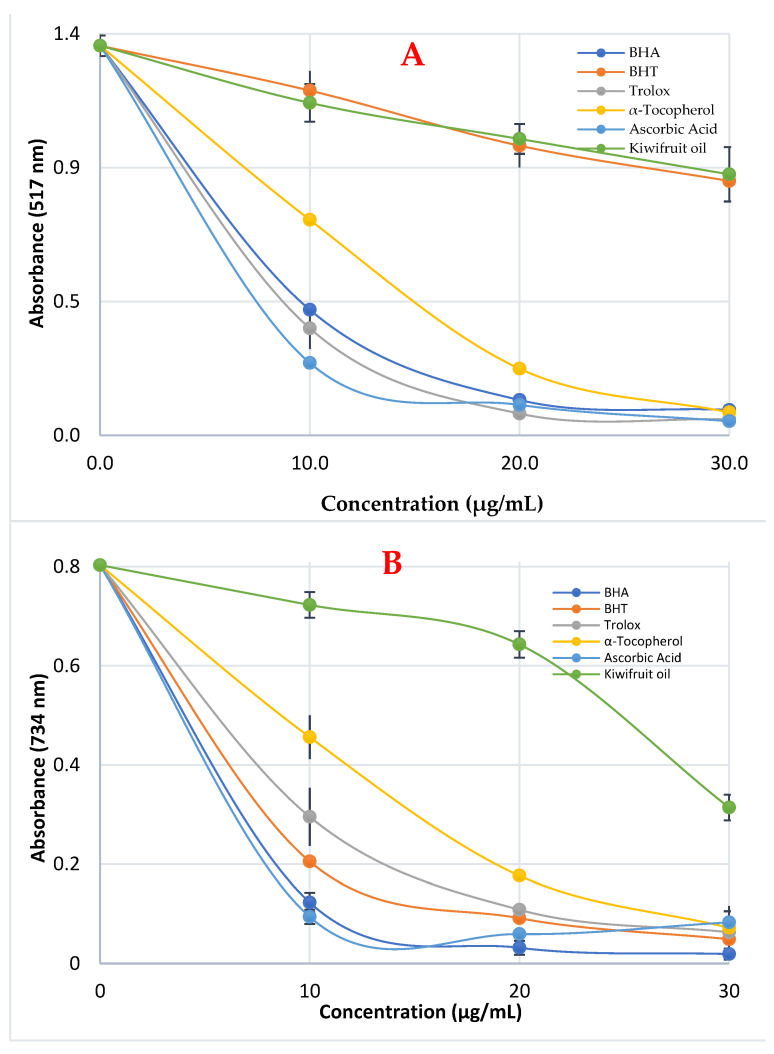
The radical scavenging effects of kiwifruit (*A. deliciosa*) oil and standards on DPPH^•^ (**A**) and ABTS^•+^ (**B**) radicals.

Kiwifruit (*A. deliciosa*) oil and standard radical scavengers have the following IC_50_ values for DPPH scavenging: 5.82 µg/mL for ascorbic acid (r^2^: 0.9668) < 6.03 µg/mL for Trolox (r^2^ = 0.9925) < 6.86 µg/mL for BHA (r^2^: 0.9949) < 7.70 µg/mL for α-tocopherol (r^2^: 0.9961) < 48.55 µg/mL for kiwifruit oil (r^2^: 0.9977) < 49.50 µg/mL (r^2^: 0.9957) for BHT. The excellent and efficient DPPH scavenging capabilities are reflected in the low IC_50_ values (see Table 4 and Figure 3A).

As shown in Figure 3B, kiwifruit (*A. deliciosa*) oil had a concentration-dependent capacity to effectively scavenge ABTS radicals (10–30 µg/mL, *p* < 0.001). Kiwifruit oil’s IC_50_ value was determined to be 77.00 µg/mL (r^2^: 0.9890) in the ABTS^·+^ clearance experiment (Table 4). The following was noted when the IC_50_ values for the reference compounds were assessed: 6.35 µg/mL for BHA (r^2^: 0.9746) < 11.74 µg/mL, for ascorbic acid (r^2^: 0.9983) < 12.60 µg/mL for BHT (r^2^: 0.9995) < 16.50 µg/mL for Trolox (r^2^: 0.9775) < 18.72 µg/mL for α-tocopherol (r^2^ = 0.9347) (Figure 3B).

### 3.4. Enzymes Inhibition by Kiwifruit Oil

Table 5 contains the findings of the inhibition for the employed enzymes. Kiwifruit (*A. deliciosa*) oil inhibited AChE with an IC_50_ value of 12.80 µg/mL oil (r^2^: 0.9680). With an IC_50_ value of 8.82 µg/mL oil (r^2^: 0.9836), the tacrine standard inhibitor suppressed AChE in the comparative study. With an IC_50_ value of 421.02 µg/mL oil (r^2^: 0.9080), as shown in Table 5, kiwifruit oil exhibited a moderately inhibitory impact on α-amylase. By inhibiting α-amylase with an IC_50_ value of 7.54 µg/mL oil (r^2^: 0.9074), the acarbose standard inhibitor was employed for comparison. Kiwifruit oil was shown to have an IC_50_ of 505.83 µg/mL oil (r^2^: 0.9249) against cytosolic and dominant hCA II isoenzyme (Table 5). With an IC_50_ value of 9.96 µg/mL oil (r^2^: 0.9930), the clinical CA isoenzyme inhibitor acetazolamide (AZA) inhibited the main and cytosolic hCA II isoforms.

## 4. Discussion

Plant phenolics are among the main classes of chemicals that operate as principal antioxidants or free radical terminators. The ability to operate as reducing agents, hydrogen atom donors and singlet oxygen scavengers makes plant polyphenols versatile. The ability to chelate transition metal ions, which may otherwise cause Fenton-type oxidation processes in their free states, makes certain polyphenols useful as antioxidants [38]. Worldwide, the use of herbal medicines as an alternative or supplement to important medical procedures is substantial. Researchers have shown a great deal of interest in developing novel medications based on the biological actions of diverse plants used in traditional or folk medicine for the treatment of diseases as a result of the rising interest in natural herbal extracts. Their medicinal benefits come from phytochemical ingredients that exhibit pharmacological features, including anticarcinogenic, antioxidant, bacterial, antiviral, and antimutagenic properties [47]. Chemicals that have antioxidant effects are found in the majority of medicinal and aromatic plants. Phenols are one of the main chemical classes present in them and have functions that include radical scavengers, metal chelators, and antioxidant. Since oxidation in biological systems is a factor in many diseases, including cancer and cardiovascular disease, fruits, vegetables, and cereals are of the utmost significance because they comprise beneficial antioxidants. Consequently, including these foods in one’s diet on a regular basis reduces the likelihood that these and other diseases will manifest. Antioxidant compounds, such as those found in plants, have the ability to slow down oxidative reactions [48].

Phenolic structures, which have a wide variety of forms and activities, are a varied category of molecules categorized as secondary metabolites in plants. For detecting phenolic chemicals and establishing a connection between them and their uses, several bioanalytical studies are available. It is anticipated that kiwifruit oil will have a high level of antioxidant activity because of its high active phenolic content. The LC-HRMS technique has the highest level of usage due to its great sensitivity. Additionally, in order to accurately determine the amount of phenol in plant-derived oils, it is crucial to optimize a number of experimental variables, including the extraction solvent, column, mobile phase, and LC-HRMS settings. The main antioxidant substances with a variety of biological effects are plant phenols, often known as polyphenols. Due to their versatility, they may operate as hydrogen atom donors, reducing agents, ROS and singlet oxygen scavengers. The design, modernization, and quality assurance of herbal medicines depend heavily on the analysis and extraction of plant material. The purpose of this research was to reveal the phytoconstituents present in the oil extract of *Actinidia deliciosa* fruits using the LC-HRMS and GC-MS techniques. The oil extract of kiwifruit includes phenolics, flavonoids and essential oil according to LC-HRMS and GCMS analysis. The chemical composition and validation parameters of *Actinidia deliciosa* oil are revealed in Table 1 and Table 2. These phenolic chemicals were shown to be the most prevalent ones in kiwifruit oil, apigenin, epigallocatechin, caryophyllene oxide, luteolin, salicylic acid, ascorbic acid, quillaic acid, rutin, and naringenin. Hepatoprotective, anti-inflammatory, antibacterial, antifungal, and antiviral properties of apigenin (4′,5,7-trihydroxyflavone) have been documented. Wines and grape skins include the flavan-3-ols catechin, epicatechin, epigallocatechin, and procyanidins, which are known for their flavor, antioxidant, and antimicrobial properties [49]. The oxidation byproduct of β-caryophyllene is caryophyllene oxide. Anticarcinogenic, anti-inflammatory, antioxidant, antiviral, and analgesic effects are some of its pharmacological activities. A powerful CNS depressant, caryophyllene oxide, also treats abnormalities of sleep and the CNS [50]. Luteolin is a flavonoid that is present in plants and is sometimes referred to as 3′,4′,5,7-tetrahydroxyflavone. In Chinese traditional medicine, various illnesses have been treated using luteolin, including hypertension, inflammatory disorders, and cancer [51]. Citric fruits, kiwi fruits, melons and leafy greens are the foods that contain ascorbic acid, a significant antioxidant that has a specific capacity to scavenge reactive nitrogen oxide species and ROS in aqueous solutions [52]. Future alternative therapeutics against diabetes inflammation and neuropathy may benefit from quillaic acid [53]. Numerous studies have demonstrated the effectiveness of a new antidiabetic medication that contains the plant flavonoids catechin, epicatechin and rutin. In addition, these compounds are potent anti-inflammatory and antioxidant molecules. An ingredient-designed study will combine them in the most effective way possible to create a new, secure, multi-target antidiabetic formulation that will be effective in managing both diabetes and its complications [54]. Citrus fruits contain large amounts of naringenin, a flavonoid with anti-atherogenic and antioxidant properties. It has been demonstrated that naringenin has antidiabetic benefits via inhibiting gluconeogenesis via AMPK activation, resulting in metformin-like actions. Naringin, like metformin, has been demonstrated to have non-glycemic effects [55].

The sole alternative to synthetic antioxidants is an antioxidant substance derived from a natural source since it is safer. These sources of naturally occurring antioxidants were thus recognized. To determine the capabilities of natural antioxidants, many techniques have been devised and are now in use. Each antioxidant assay examines antioxidant activity using a distinct mechanism of action; for example, the FRAP assay only evaluates the antioxidant’s capacity to transfer a single electron, whereas the DPPH assay evaluates both hydrogen atom and electron transfer [38]. For this purpose, several antioxidant techniques were conducted to determine the kiwifruit oil’s antioxidant capabilities. Plant-derived oils’ biological activities are facilitated by their potential for reduction. These oils lessen oxidative stress and neutralize ROS because of their strong reducing ability [38]. The reduction potential of oils or extracts obtained from plants may be directly assessed using the Fe^3+^ reduction technique. Under experimental circumstances, the blue-colored Fe_4_[Fe(CN^–^)_6_]_3_ that is formed when kiwifruit oil is added to solutions containing Fe^3+^ ions may absorb light at a wavelength of 700 nm. In addition to the development of this chromophore complex, the color of the test combination demonstrates the plant extracts’ capacity to reduce in a range of the yellow to green color spectrum [56]. In the reduction experiments for Fe_4_[Fe(CN^−^)_6_]_3_, Fe^3+^-TPTZ, and Cu^2+^ kiwifruit oil showed a moderately efficient reducing ability. The findings show that the e-donor property of kiwifruit oil effectively counteracts the negative effects of ROS and free radicals. The reductive potential of this compound was comparable to that of α-tocopherol and ascorbic acid, but it was less than that of BHT, Trolox and BHA. For plant extracts and oils, the CUPRAC technique employed in his research is an inexpensive, discerning, reliable, and quick procedure that is unaffected by chemical components and hydrophobicity. The FRAP reduction method was the last reduction test employed in this investigation. Strong absorbance results, just like in previous reduction studies, indicate that the compound has a strong reduction potential. To maintain the solubility of Fe^3+^ ions, the FRAP technique should also be used in acidic environments [57].

In general, it is accepted that phenolic chemicals and flavonoids help plants achieve their full antioxidant potential. Therefore, several studies have demonstrated that the total flavonoid or total phenolic ingredients of plants strongly correlate with their antioxidant potential. Establishing the in vitro antioxidant capacity of plant materials does, in fact, frequently call for numerous experiments [58]. An ingredient’s capacity to scavenge free radicals reveals how antioxidant and capable it is of preventing the start of an oxidation chain. The ability of a chemical to scavenge radicals has often been assessed using the DPPH and ABTS tests [37]. The ABTS^•+^ and DPPH^•^ removal tests were used to determine the kiwifruit oil’s antioxidant capabilities. They are the best and most popular radical removal tests. They are utilized to assess the radical scavenging capabilities of substances. *Actinidia deliciosa* L. methanolic extract had 68.236% more DPPH-scavenging activity (IC_50_) at 250 ppm compared to ascorbic acid, which had 81.262% more activity. Ascorbic acid, the reference drug, had an IC_50_ of 166.093 ± 2.6 ppm, whereas methanolic extract had an IC_50_ of 189.170 ± 3.2 ppm [59]. Actinidia fruits were examined in a different research using the DPPH and ABTS assays. The TEAC values for the DPPH and ABTS tests were determined to be 0.11 to 0.49 µM Trolox Equivalent (TE)/g FW and 8.04 to 14.39 µM TE/g FW, respectively [60]. The values found when using DPPH ranged from 952 ± 78 to 3115 ± 35 M trolox, while those determined using ABTS ranged from 346 ± 20 to 1651 ± 3 M trolox for free radical scavenging activity. *A. eriantha* and *A. latifolia* had more antioxidant capacity in the DPPH and ABTS techniques than other genotypes of Actinidia fruit; however, *A. deliciosa* Cv. Hayward had a lower antioxidant capacity [61]. The DPPH experiment revealed that the different kiwifruit had an antioxidant capacity ranging from 75.07 to 327.96 mg ascorbic acid equivalent (AAE) at 100 g^−1^ fresh weight (FW). According to the results of the ABTS experiment, there were considerable differences in the antioxidant capacity of the different kiwifruit, ranging from 95.37 mg AAE at 100 g^−1^ FW to 474.59 mg AAE at 100 g^−1^ FW [62]. The rejected kiwifruit is a great source of antioxidants since it contains a lot of phenolic components. In comparison to Hayward and round organic Hayward, the Sun Gold variety, in particular, demonstrated the best TPC result and the strongest antioxidant capacity in DPPH, FRAP, and ABTS assays [63]. The results of a study showed that an aqueous extract of *A. deliciosa* (1 g/kg orally) effectively prevents streptozotocin (STZ; 50 mg/kg, i.p., single dose)-induced diabetic nephropathy in rats by preventing oxidative stress, scavenging free radicals, reducing inflammation, and reestablishing endogenous antioxidant defense system mechanisms [64]. In a prior study conducted at the Gulcin laboratory, the lyophilized aqueous extract of kiwifruit (*A. deliciosa*) was shown to have IC_50_ (for radical scavenging test) values against ABTS^•+^ and DPPH^•^ scavenging activities were assessed as 28.4 ± 2.14 and 83.4 ± 4.67 g/mL, respectively [65]. At a concentration of 45 µg/mL, the EC_50_ value of clove oil was determined to be 21.50 µg/mL, 14.83 µg/mL on DPPH and ABTS radical scavenging activity, in the given order [66]. The antioxidant capacities of two kiwifruit varieties were compared. The antioxidant capacities of the cv. Bidan kiwifruit were measured with the ABTS and DPPH assays, and they were 608.9 mg AAE/100 g fresh weight and 620.9 mg AAE/100 g fresh weight, respectively. The antioxidant capacities of the cv. Hayward kiwifruit were 143.5 and 116.0 mg AAE/100 g fresh weight, according to the given order. Compared to cv. Hayward kiwifruit, the antioxidant capacity of cv. Bidan kiwifruit was around 2.4–5.4 times greater [67]. Based on their maturation stages, six kiwifruit cultivars produced in Kore were assessed. It demonstrates the antioxidant capabilities of the six kiwifruit cultivars at various stages of maturity, as determined using ABTS and DPPH tests. Depending on the stage of maturity, each cultivar had varying degrees of antioxidant capacity. According to the results of the ABTS experiment, the antioxidant capacity of the flesh of different kiwifruits ranged from 99.0 mg AAE 100 g^−1^ FW in ripe “Chiak” to 816.5 mg AAE 100 g^−1^ FW in Bidan harvested in the second harvest stage. The antioxidant capabilities of different kiwifruit flesh were estimated using the DPPH test to be between 46.0 and 633.2 mg AAE 100 g^−1^ FW [68]. Regardless of the quantity ingested, kiwifruit consumption boosted antioxidant levels in healthy individuals and offered protection against DNA oxidative damage. In total, 14 healthy participants (eight women and six men, ages 26 to 54) received antioxidant protection from kiwifruit eating (1–3 per day, 3 weeks), as evaluated in terms of lymphocytes [69]. *A. chinensis* ‘Hort 16A’ was added to the typical diet of 24 healthy volunteers (men and women, 20 to 57 years old, with BMIs of 20 to 30 kg/m^2^) for four weeks. This reduced the H_2_O_2_-induced DNA detriment and boosted plasma levels of ascorbic acid. The consumption of golden kiwifruit on a regular basis can boost plasma ascorbic acid levels and prevent the oxidation of lymphocyte DNA. A regular intake can also lessen plasma triglycerides and platelet aggregation [70]. Our kiwifruit (*A. deliciosa*) results also have valuable and efficient antioxidant (ABTS^•+^ scavenging and DPPH^•^ scavenging activities) and reducing power (Fe^3+^, Cu^2+^ and FRAP) properties when compared with the findings of all studies carried out previously.

In studies on drug production and design, the procedure of enzyme inhibition is a popular and successful method. The symptoms of numerous illnesses that involve obesity, diabetes, and diabetes-related disorders can be reduced by inhibiting certain metabolic enzymes. Some synthetic inhibitors have been documented to have negative effects, such as gastrointestinal problems and hepatotoxicity. Finding new and natural inhibitors without adverse effects from natural compounds is thus of tremendous interest [71]. In this research, it was discovered that kiwifruit oil effectively inhibits AChE, α-amylase, and hCA II, which are linked to common metabolic illnesses, including AD and T2DM, when the findings of the enzyme inhibition experiments were reviewed and compared with those of conventional inhibitors.

The previous study’s findings did show, however, that the sort of extraction procedure utilized can affect oregano’s ability to inhibit cholinesterase. Particularly when oregano leaves were used, AChE inhibition was shown to be more potent, whereas water extracts showed less inhibition [72]. Both *Origanum ehrenbergii* and *Origanum syriacum* oils had an intriguing inhibitory action on AchE and BchE, important enzymes in the pathogenesis of AD, even at extremely low doses [23]. The breakdown of acetylcholine (a neurotransmitter) is catalyzed by acetylcholinesterase and butyrylcholinesterase, as demonstrated in another research by freeze-dried kiwifruits. Among the freeze-dried kiwifruits, cv. Happygold scored best on the sensory evaluation for overall preference. According to the findings, kiwifruit that has been freeze-dried may be a rich source of cholinesterase inhibitors and antioxidants [73]. As a functioning cholinesterase inhibitor, kiwis may be a useful source of antioxidants. These findings indicated that kiwifruit oil, when compared to tacrine, was a potent and appropriate AChE inhibitor.

In particular, for postprandial glucose level (PPGL) and T2DM, natural products’ inhibition of α-amylase can successfully lower blood glucose levels. The typical inhibitor acarbose had a stronger inhibitory action than kiwifruit oil (IC_50_: 22.800 mM) [74]. Different research used an in vitro digestion technique to separate soluble and insoluble dietary fibers (SDF and IDF) from ripe kiwifruit to examine if they have an inhibitory effect on the enzyme α-amylase. Initial starch hydrolysis velocity was shown to be dose-dependently reduced in both IDF and SDF. IDF had a greater α-amylase inhibitory action when applied at the same concentration [75]. Fourteen distinct varieties of kiwifruit imported from China were rigorously examined in order to find high-quality fruit with health-enhancing qualities. All of the examined kiwifruits also showed impressive antioxidant properties and α-glucosidase and pancreatic lipase inhibitory activities [76]. In this research, we observed another diabetes regulator enzyme, α-amylase enzyme inhibition, using kiwifruit oil, which can regulate and bring the destabilized levels of glucose to a steady state. Blood glucose levels may be lowered as a result of the digestive enzymes’ inhibitory actions, particularly on α-amylase. Also, its inhibition could significantly improve diabetes-related hyperglycemia control. Research continuing all over the world has shown that medicinal plants may effectively treat diabetes and its associated consequences.

Since phenolic substances are a bit acidic, they lose protons (H^+^) from their hydroxyl groups and transform into extremely soluble phenolate anions in water. Because their scaffolds contain functional groups such as phenolic -OH, -OCH_3_, and -COOH groups, it is well-known that phenolic compounds may effectively block CA isoenzymes [77]. A CA inhibition mechanism is present in several of the examined phenols. In the active site cavity, phenols bind more externally and interact with different amino acid residues after being anchored on the Zn^2+^-coordinated water molecule. This class of chemicals may result in isoform-selective inhibitors that target only a few of the medicinally important CAs, as this area has the largest diversity among the many CA isozymes discovered in mammals [42]. Kiwifruit’s various extracts have not been studied in CA II inhibition before; it is open to research. Our study may be a pioneer for future studies.

## 5. Conclusions

Kiwifruit stands to be one of the most widely consumed fruits globally, valued for its delightful taste and array of health benefits. It is commonly believed that its proven biological activity lends protection against various illnesses when included in the diet. This fruit surpasses many commonly eaten fruits in terms of phenolic acids. The antioxidant properties of kiwifruit were investigated using numerous in vitro bioanalytical analyses, and effective results were obtained. For example, kiwifruit’s oil effectively demonstrated DPPH radical scavenging (IC_50_: 48.55 μg/mL) and ABTS radical scavenging (IC_50_: 77.00 μg/mL). Also, its inhibitory effects on key enzymes, including AChE (IC_50_: 12.80 μg/mL), α-amylase (IC_50_: 421.02 μg/mL) and CA II (IC_50_: 505.83 μg/mL) associated with diabetes, glaucoma and Alzheimer’s disease are extremely important. The study also aimed to identify potentially active compounds present in kiwifruit oil. Utilizing LC-HR/MS analysis unveiled phenolic compounds such as apigenin (74.24 mg/L oil), epigallocatechin (12.89 mg/L oil), caryophyllene oxide (12.89 mg/L oil), and luteolin (5.49 mg/L oil) in kiwifruit oil. Furthermore, the GC-FID analysis of kiwifruit oil highlighted squalene as the dominant component, constituting 53.04% of the overall oil composition. The oil also harbored a rich assortment of naturally occurring phenolic compounds, with linoleoyl chloride, linoleic acid, and palmitic acid being the most prevalent. The findings strongly indicate that kiwifruit oil can serve as a valuable source of essential biomolecules that are crucial for biological functions. The presence of polyphenolic compounds and ascorbic acid in kiwifruit oil, known for their antioxidant, antidiabetic, and anti-Alzheimer effects, contribute significantly to disease prevention and overall well-being.

## Figures and Tables

**Table 1:** Chemical composition and validation parameters of kiwifruit (*A. deliciosa*) (mg/L oil) obtained using LC-HRMS.

Phenolic Compounds	Molecular Formula	*m*/*z*	Ionization Mode	Linear Range	Linear Regression Equation	LOD/LOQ	R²	Recovery	U%	Phenolics
Ascorbic acid	C_6_H_8_O_6_	175.0248	Negative	0.5–10	y = 0.00347x − 0.00137	0.39/1.29	0.9988	96.20	3.94	4.57
Epigallocatechin	C_15_H_14_O_7_	307.0812	Positive	0.3–5	y = 0.00317x + 0.000443	0.17/0.57	0.9947	102.22	3.09	12.89
Chlorogenic acid	C_16_H_18_O_9_	353.0878	Negative	0.05–10	y = 0.00817x + 0.000163	0.02/0.06	0.9994	96.68	3.58	0.61
Fumaric acid	C_4_H_4_O_4_	115.0037	Negative	0.1–10	y = 0.00061x − 0.0000329	0.05/0.17	0.9991	97.13	2.88	<LOD
Verbascoside	C_29_H_36_O_15_	623.1981	Negative	0.1–10	y = 0.00758x + 0.000563	0.03/0.1	0.9995	96.19	2.93	0.25
Orientin	C_21_H_20_O_11_	447.0933	Negative	0.1–10	y = 0.00757x + 0.000347	0.01/0.03	0.9993	96.22	3.67	0.43
Caffeic acid	C_9_H_8_O_4_	179.0350	Negative	0.3–10	y = 0.0304x + 0.00366	0.08/0.27	0.9993	94.51	3.74	0.38
Luteolin-7-rutinoside	C_27_H_30_O_15_	593.1512	Negative	0.1–10	y = 0.00879x + 0.000739	0.01/0.03	0.9988	93.05	3.06	0.27
Luteolin-7-glycoside	C_21_H_20_O_11_	447.0933	Negative	0.1–7	y = 0.0162x + 0.00226	0.01/0.03	0.9961	96.31	4.14	0.48
Rutin	C_27_H_30_O_16_	609.1461	Negative	0.05–10	y = 0.00329x − 0.00005576	0.01/0.03	0.999	96.97	3.07	4.54
Rosmarinic acid	C_18_H_16_O_8_	359.0772	Negative	0.05–10	y = 0.00717x − 0.0003067	0.01/0.03	0.9992	99.85	3.77	0.19
Hyperoside	C_21_H_20_O_12_	463.0882	Negative	0.05–10	y = 0.0072x − 0.00003096	0.01/0.03	0.9995	96.62	3.46	0.45
Apigenin 7-glycoside	C_21_H_20_O_10_	431.0984	Negative	0.3–7	y = 0.0246x + 0.00306	0.01/0.03	0.9962	96.07	2.86	0.93
Ellagic acid	C_14_H_6_O_8_	300.9990	Negative	0.05–10	y = 0.0085x − 0.000612	0.03/1	0.9994	101.49	3.59	0.04
Quercitrin	C_21_H_20_O_11_	447.0933	Negative	0.05–10	y = 0.0179 + 0.0003331	0.01/0.03	0.999	97.00	4.20	0.10
Quercetin	C_15_H_10_O_7_	301.0354	Negative	0.1–10	y = 0.0509x + 0.00467	0.01/0.03	0.9978	96.41	3.78	2.36
Herniarin	C_10_H_8_O_3_	177.0546	Positive	0.1–7	y = 0.309x + 0.0266	0.01/0.03	0.9983	92.92	2.95	1.44
Salicylic acid	C_7_H_6_O_3_	137.0244	Negative	0.3–10	y = 0.0361x + 0.00245	0.01/0.03	0.9982	92.88	3.89	4.84
Naringenin	C_15_H_12_O_5_	271.0612	Negative	0.1–10	y = 0.0281x + 0.00182	0.01/0.03	0.9995	86.65	1.89	3.62
Luteolin	C_15_H_10_O_6_	285.0405	Negative	0.1–10	y = 0.117x + 0.00848	0.01/0.03	0.9981	96.98	4.20	5.49
Apigenin	C_15_H_10_O_5_	269.0456	Negative	0.3–10	y = 0.104x + 0.0199	0.01/0.03	0.9998	81.55	3.42	74.24
Hispidulin	C_16_H_12_O_6_	301.0707	Positive	0.05–10	y = 0.02614x + 0.0003114	0.01/0.03	0.9993	98.36	2.87	1.99
Isosakuranetin	C_16_H_14_O_5_	285.0769	Negative	0.05–10	y = 0.0235x + 0.000561	0.01/0.03	0.9992	96.56	3.41	0.08
Penduletin	C_18_H_16_O_7_	343.0823	Negative	0.3–10	y = 0.0258x + 0.00253	0.01/0.03	0.9991	83.43	3.20	1.31
CAPE	C_17_H_16_O_4_	283.0976	Negative	0.3–7	y = 0.255x + 0.0477	0.01/0.03	0.9964	94.42	3.13	2.17
Chrysin	C_15_H_10_O_4_	253.0506	Negative	0.05–7	y = 0.0964x − 0.0002622	0.01/0.03	0.999	87.92	3.24	>LOQ
Quillaic acid	C_30_H_46_O_5_	485.3273	Negative	0.05–10	y = 0.00781x − 0.0001318	0.01/0.03	0.9992	90.29	2.56	4.57
Caryophyllene oxide	C_15_H_24_O	221.1900	Positive	0.3–7	y = 0.00151x + 0.00692	0.10/0.50	0.9909	96.87	4.05	12.89

**Table 2:** The chemical composition of essential oil was derived from kiwifruit (*A. deliciosa*) oil using GC-MS.

Essential Oils	RT (min)	Formula	Contents (%)
Cetal	16.15	C_17_H_34_O	0.91
Stenol	19.83	C_18_H_38_O	0.97
Palmitic acid	22.65	C_16_H_32_O_2_	1.54
Linoleic acid	25.37	C_18_H_32_O_2_	2.67
Linoleoyl chloride	25.47	C_18_H_31_ClO	20.28
Squalene	34.52	C_30_H_50_	53.04
Total			79.41

**Table 3:** Ferric ions (Fe^3+^), cupric ions (Cu^2+^), and Fe^3+^-TPTZ complex-reducing ability of kiwifruit (*A. deliciosa*) oil and standards at 30 μg/mL concentration (BHA: butylated hydroxyanisole, BHT: butylated hydroxytoluene).

Antioxidants	Fe^3+^ Reducing *		Cu^2+^ Reducing *		Fe^3+^-TPTZ Reducing *	
	λ_700_	r^2^	λ_450_	r^2^	λ_593_	r^2^
BHA	2.292 ± 0.012	0.9993	2.418 ± 0.018	0.9887	1.172 ± 0.014	0.9605
BHT	2.136 ± 0.090	0.9957	1.953 ± 0.045	0.9998	0.690 ± 0.008	0.9645
Trolox	1.514 ± 0.066	0.9963	1.800 ± 0.096	0.9974	1.180 ± 0.032	0.9732
α-Tocopherol	0.862 ± 0.038	0.9996	0.851 ± 0.046	0.9994	0.918 ± 0.011	0.9904
Ascorbic acid	2.298 ± 0.086	0.9659	0.983 ± 0.048	0.9822	1.257 ± 0.024	0.9869
Kiwifruit oil	0.835 ± 0.035	0.9723	0.765 ± 0.031	0.9978	0.583 ± 0.017	0.9525

* All results are shown as mean ± SD and are the averages of three parallel observations (n = 3).

**Table 4:** IC_50_ values (μg/mL) of the kiwifruit (*A. deliciosa*) oil and standards for the scavenging of DPPH^•^ and ABTS^•+^ radicals.

Antioxidants	DPPH^•^ Scavenging		ABTS^•+^ Scavenging	
	IC_50_	r^2^	IC_50_	r^2^
BHA	6.86	0.9949	6.35	0.9746
BHT	49.50	0.9957	12.60	0.9995
Trolox	6.03	0.9925	16.50	0.9775
α-Tocopherol	7.70	0.9961	18.72	0.9347
Ascorbic acid	5.82	0.9668	11.74	0.9983
Kiwifruit oil	48.55	0.9977	77.00	0.9890

**Table 5:** The IC_50_ values (µg/mL oil) of kiwifruit (*A. deliciosa*) oil towards α-amylase, acetylcholinesterase, and carbonic anhydrase II enzymes.

Enzymes	Kiwifruit (*A. deliciosa*) Oil		Standard Inhibitors	
	IC_50_	r^2^	IC_50_	r^2^
α-Amylase ^1^	421.02	0.9080	7.54	0.9074
Acetylcholinesterase ^2^	12.80	0.9680	8.82	0.9836
Carbonic anhydrase II ^3^	505.83	0.9249	9.96	0.9930

^1^ Acarbose was employed as a positive control for the enzymes α-glycosidase and α-amylase. ^2^ Acetylcholinesterase enzyme was tested using tacrine as a positive control. ^3^ A positive control for carbonic anhydrase II isoenzyme was Acetazolamide (AZA).

## Data Availability

Data are available in a publicly accessible repository.
